# Machine learning for predicting thrombotic recurrence in antiphospholipid syndrome

**DOI:** 10.1016/j.rpth.2025.103198

**Published:** 2025-09-30

**Authors:** Ana Marco-Rico, Ihosvany Fernández-Bello, Jorge Mateo-Sotos, Pascual Marco-Vera

**Affiliations:** 1 Hospital General Universitario Dr. Balmis, Department of Hematology, Instituto de Investigación Sanitaria y Biomédica de Alicante, Alicante, Spain; 2 Institute of Technology, Medical Analysis Expert Group, Universidad de Castilla-La Mancha, Cuenca, Spain; 3 Department of Clinical Medicine, Universidad Miguel Hernández, Alicante, Spain

**Keywords:** antiphospholipid syndrome, machine learning, thrombosis, recurrence, risk assessment

## Abstract

**Background:**

Thrombotic antiphospholipid syndrome (TAPS) is an autoimmune disorder associated with a high risk of recurrent thromboembolic events. Despite advances in anticoagulation, predicting recurrence remains challenging, underscoring the need for more precise risk stratification to optimize personalized treatment. Traditional predictive models struggle to integrate the complexity of clinical and biochemical risk factors, creating an opportunity for machine learning to enhance prognostic accuracy.

**Objective:**

In this study, we evaluated the performance of the extreme gradient boosting (XGB) model in predicting recurrent thrombotic events in TAPS, compared with other machine learning algorithms.

**Methods:**

Demographic and clinical data were initially included, and model performance was assessed through multiple metrics, such as accuracy, specificity, precision, and the area under the receiver operating characteristic curve.

**Results:**

XGB outperformed all other models, achieving the highest area under the receiver operating characteristic curve and accuracy, among other evaluated parameters, demonstrating robust predictive capabilities. Key predictors included renal impairment, age, and the presence of lupus anticoagulant, reinforcing the clinical relevance of these factors in risk assessment.

**Conclusion:**

These findings highlight the potential of XGB to improve risk stratification and support clinical decision-making in TAPS. By identifying critical predictors, this approach may optimize anticoagulation strategies and enhance resource allocation. However, further validation in larger cohorts and prospective studies is necessary before clinical integration.

## Essentials

• Novel methodologies for predicting an accurate thrombotic recurrence in antiphospholipid syndrome are needed.
• Artificial intelligence can predict thrombotic recurrence in patients with antiphospholipid syndrome.
• Renal insufficiency, age, and positive lupus anticoagulant are the main key predictors of thrombotic recurrence.
• Machine learning algorithms enable personalized risk stratification and optimize anticoagulation strategies.

## 1 Introduction

Antiphospholipid syndrome (APS) is an autoimmune disorder characterized by thrombosis (venous, arterial, or small vessels) or adverse pregnancy outcome (≥3 consecutive miscarriages before week 10 of gestation, ≥1 miscarriage after week 10 or premature birth before of week 34 in the context of eclampsia, preeclampsia, or placental insufficiency) in the presence of persistent antiphospholipid antibodies (aPL) [1]. Therefore, APS represents a heterogeneous condition with a wide spectrum of clinical manifestations, ranging from isolated thrombotic events to obstetric complications, and in severe cases, catastrophic presentations involving multiorgan failure may appear [2]. APS can occur as a primary condition or in the setting of autoimmune diseases like systemic lupus erythematosus [3]. Thrombotic APS (TAPS) predominantly affects venous territories, and deep vein thrombosis of the lower limbs is the most common clinical presentation [4]. Other locations, including pulmonary embolism, portal vein thrombosis, and ischemic strokes are also frequently observed [5]. The risk of recurrence is strongly influenced by the presence of aPL, lupus anticoagulant (LAC) being the most pathogenic, followed by anti-β_2_-glycoprotein I antibodies (anti-β_2_-GPI) immunoglobulin (Ig) G and anticardiolipin (aCL) IgG [6,7]. Anti-prothrombin antibodies, not included in European Alliance of Associations for Rheumatology APS criteria, have been associated with a higher rate of thrombosis compared with negative anti-prothrombin patients (8.6 vs 3.6 per patient-year) [8]. In addition, patients presenting with LAC, aCL, and anti-β_2_-GPI are considered triple-positive APS [9]. These patients have the highest risk for thrombosis, reaching a relative risk of 33, compared with single or double combinations of the mentioned antibodies [10]. Owing to the high recurrence rate, even on anticoagulation, indefinite antithrombotic treatment remains the treatment of choice in APS [11]. Crowther et al. [12] included 114 patients with APS (74% with venous TAPS) and were followed for a median of 2.7 years. In patients receiving vitamin K antagonists (VKA), thrombotic recurrence occurred in 10.7% patients with high-intensity anticoagulation versus 3.4% in the standard anticoagulation group (hazard ratio [HR], 3.1; 95% CI, 0.6-15). The bleeding risk was similar in both groups (5.4% vs 6.9%, respectively; HR, 1; 95% CI, 0.2-4.8) [12]. Finnazi et al. [13] reported similar recurrence thrombotic rates (11.1% with high-intensity anticoagulation vs 5.5% in case of standard intensity anticoagulation (HR, 1.97; 95% CI, 0.47-7.89), and the bleeding risk did not reach statistical significance (27.8% vs 14.6%; HR, 2.18; 95% CI, 0.92-5.15). In 2017, another study reported that recurrence rates in APS patients treated with anticoagulants reached 23.7%, compared with 37.2% in those on antiplatelet therapy and 6.9% in those receiving combined therapy, with an estimated 20% recurrence occurring within 3.4, 7.3, and 16.3 years, respectively [14]. More recently, in 2018, a meta-analysis of APS patients treated with direct oral anticoagulants (DOACs) reported a recurrence rate of 16%, with a mean duration of 12.5 months until the next thrombotic event, underscoring the limitations of DOACs compared with traditional anticoagulants [15]. Given these challenges, it is urgently needed to find new methodologies to achieve the most accurate possible prediction of thrombosis recurrence in this population. However, traditional statistical methods for predicting thrombotic recurrence in APS are limited due to their inability to capture the multifactorial and the non-linear nature of APS [16]. The heterogeneity of APS, including variations in clinical presentation, antibody profiles, and response to therapy, highlights the inadequacy of conventional models for stratifying thrombotic risk.

Machine learning (ML) algorithms have emerged as transformative tools in medicine, offering predictive capabilities that surpass traditional methods by integrating complex, multidimensional datasets, including clinical, demographic, and laboratory variables [17]. ML has the potential to modify patients’ outcomes, enhance the quality of care and, even more importantly, make healthcare more accessible and cost-effective. The benefits of artificial intelligence (AI) in clinical practice include rapid processing of large-scale data, correlation analysis to remove redundant variables, avoiding overfitting and the use of objective and consistent criteria to enhance diagnostic accuracy and specificity [18]. Unlike conventional approaches, ML models are very promising for identifying complex patterns and interactions and serve as a powerful tool for thrombotic risk assessment, making them particularly well-suited for risk predictions in APS [19]. APS diagnosis can be challenging, and data with AI are scarce. De Laat et al. [20] used an artificial neural network (NN) that can diagnose APS in VKA-treated patients, based on thrombin generation. Five NNs were trained to diagnose APS in 48 VKA-treated APS patients and 64 VKA-treated controls. Two best-performing NNs were selected (accuracy of 96%; sensitivity 96%-98%; and specificity 95%-97%) and validated in an independent cohort of VKA anticoagulated APS patients (*n* = 33) and controls (*n* = 62). Independent clinical validation favored one of the 2 selected NNs, with a sensitivity of 88% and a specificity of 94% for the diagnosis of APS. In conclusion, the combined use of thrombin generation and NN methodology diagnosed APS with an accuracy of 92% [20].

By addressing the limitations of conventional analysis, the objective of this study was to assess ML algorithms for predicting recurrence risk in individuals with TAPS on anticoagulation.

## 2 Material and Methods

### 2.1 Study Design

We designed a cross-sectional study including patients with TAPS on anticoagulation followed at the Thrombosis and Hemostasis Unit of Hospital General Universitario Dr Balmis in Alicante, Spain. This study was conducted after receiving approval from the ethics committee of the Hospital Universitario Dr Balmis (Alicante, Spain). The study was accomplished in accordance with the basic principles of the World Medical Association Declaration of Helsinki and complied with the standards described in the European Union Guidelines for good clinical practice.

### 2.2 Inclusion criteria

Male and female patients aged ≥18 years with a diagnosis of TAPS, receiving antithrombotic treatment, were eligible for the study. All patients provided signed informed consent prior to inclusion.

### 2.3 Exclusion criteria

Patients with incomplete clinical data were excluded. Additional exclusion criteria comprised the use of intensive immunosuppressive therapies that could interfere with the interpretation of thrombotic recurrence, documented poor treatment adherence, discontinuation of anticoagulant therapy during the follow-up period, end-stage renal disease requiring dialysis, pregnant women, and active cancer.

### 2.4 Clinical data

Variables were selected based on their known relevance to thrombotic risk in patients with TAPS. Data were extracted from an anonymized institutional database and included clinical, laboratory, and treatment-related variables documented during routine follow-up. Demographic characteristics (age and sex [biological male or female]), common cardiovascular risk factors (including hypertension, diabetes mellitus, dyslipidemia, renal insufficiency, obesity, and smoking status), and the presence of autoimmune conditions were recorded. Thrombosis-related variables encompassed the location of the first thrombotic event (venous, arterial, or small vessel) and thrombotic recurrence during follow-up.

### 2.5 Laboratory data

Laboratory parameters included the LAC (expressed as a ratio between screening and confirm ratio), aCL (IgG and IgM, in IU/mL), anti-β_2_-GPI (IgG and IgM in IU/mL), and D-dimer levels (ng/mL). LAC was confirmed by 2 techniques with different mechanisms of action, in agreement with the latest ISTH (International Society on Thrombosis and Hemostasis) guidelines, in particular, by dilute Russell viper venom time (Staclot Dilute Russell’s Viper Venom Time screening and confirm, Diagnostica Stago) and the use of silica as a reagent sensitive to LAC derived from activated partial thromboplastin time (Silica, Diagnostica Stago) following manufacturer’s protocols. LAC was considered positive if the ratio >1.30. APL were analyzed by chemiluminescence (HemosIL AcuStar) and were positive if the result was > 20 IU/mL, and D-dimer levels were determined by immunoprecipitation (STA-Liatest D-Di, Diagnostica Stago), in agreement with manufacturer’s instructions.

Renal insufficiency was considered if glomerular filtration was < 30 mL/min/1.73m^2^ and obesity if body mass index (BMI) > 30 kg/m^2^. The following thrombophilia parameters were recorded: factor (F)V Leiden and PT210A mutation, methylentetrahydrofolate reductase mutation, protein S and C deficiency, and antithrombin deficiency.

Antithrombotic treatments were categorized into VKA, low-molecular-weight heparin, DOACs, and antiplatelet agents. For patients on VKA, the last value of the international normalized ratio (INR) was recorded and dichotomized as 1 if INR >60% of the upper limit of the normal range, otherwise 0. Patients with APS and a first thrombotic episode had a target INR range from 2 to 3.

### 2.6 Predictive ML modeling and data analysis

In this study, the extreme gradient boosting (XGB) algorithm was implemented to predict thrombotic recurrence in patients with APS, and its performance was compared with 5 reference algorithms: Random Forest, Support Vector Machine, Decision Tree, Gaussian Naïve Bayes, and K-nearest neighbors, to verify whether a regulated ensemble model outperforms approximations based on margins, distance, or probability. To homogenize scales and facilitate the convergence of the classifiers, continuous variables were transformed into z-scores, while highly collinear predictors (r > 0.80) were refined to avoid redundancy and reduce the risk of overfitting. Missing values were addressed within the nested cross-validation (CV) pipeline to prevent information leakage. Continuous features were imputed using the median, and categorical features using the mode, calculated exclusively from the training folds. The imputation procedure was re-applied independently for each CV split, ensuring that the test folds remained unseen. No complete case analysis was performed, as this would have led to unnecessary sample loss and potential selection bias. In all cases, the proportion of missing data per variable was <5%. Continuous variables were compared using the Mann Whitney *U* test, while categorical variables were analyzed using the chi-squared test or Fisher’s exact test, as appropriate. Given the limited sample size, multiple strategies to minimize overfitting were implemented. Model evaluation was conducted using nested, stratified CV, with a 5-fold outer loop for unbiased performance estimation and an inner loop for hyperparameter optimization. The full nested CV procedure was repeated 200 times with different random seeds to reduce variability associated with data partitioning. For XGB, the hyperparameter search space was deliberately constrained to limit effective capacity: max_depth ≤4, min_child_weight ≥2, subsample ≤0.8, colsample_bytree ≤0.8, and a tuned learning_rate from 0.02 to 0.30. This approach reduces the likelihood of fitting noise while preserving the ability to capture clinically relevant patterns. All preprocessing steps (imputation, scaling, and encoding) were confined to the training folds only, preventing information leakage. Final performance estimates were derived by averaging across all repetitions, with 95% CIs calculated via bootstrapping of outer-loop predictions. The recurrence group accounted for 30.6% of the study cohort. To address this imbalance, synthetic oversampling was applied within the training folds of each nested CV iteration using the Synthetic Minority Oversampling Technique (SMOTE), following principles for preserving inter-feature correlations during data synthesis [21]. The algorithm interpolated new minority-class cases from 5 nearest neighbors until equal representation of classes was achieved. Importantly, resampling was performed after imputation and scaling had been fitted on the training partition, thereby preventing information leakage into the test folds. To evaluate the robustness of this approach, additional analyses incorporated alternative imbalance-handling strategies, including Borderline-SMOTE, SMOTEENN, and cost-sensitive learning by adjusting the scale_pos_weight parameter of XGB to the observed class ratio (Supplementary Table 1). The XGB model was optimized within the inner loop of the nested CV, with the final hyperparameters set as: max_depth = 4, min_child_weight = 2, subsample = 0.8, colsample_bytree = 0.8, learning_rate = 0.05, n_estimators = 300, gamma = 0, and scale_pos_weight ≈ 2.27. Continuous predictors were winsorized at the 1st and 99th percentiles and standardized to z-scores using statistics from the training folds only. Categorical predictors were one-hot encoded, with missing values imputed by the mode; missing continuous values were imputed by the median. All preprocessing was conducted within folds to prevent information leakage. Collinearity was addressed by clustering highly correlated predictors (|r| > 0.80) and retaining a single representative per cluster based on clinical interpretability, lower missingness, and lower variance inflation. After refinement, all variables exhibited variance inflation factor <5.

The primary metrics selected were the area under the receiver operating characteristic curve (AUC-ROC), the F1 score, and the Mathews correlation coefficient, as they combine information on overall discrimination, the balance between false positives and false negatives, and classifier quality in unbalanced contexts. Secondary metrics reported were sensitivity, accuracy, specificity, positive precision, and the Youden index, which are useful for nuanced interpretation but susceptible to bias when one class dominates [[22], [23], [24]].

To assess model calibration, we evaluated the Brier score, which is the average of the squared difference between the model-based prediction and actual outcome (0 = no thrombotic recurrence; 1: thrombotic recurrence).

A *P* < .05 was considered statistically significant. All analyses were performed using Python (version 3.11; Python Software Foundation), or SPSS (IBM SPSS version 26 (IBM Corp), with appropriate statistical libraries. The primary libraries used were: xgboost v1.7.x (developed by the XGBoost Developers distributed by the DMLC Team), scikit-learn v1.4.x (developed by the scikit-learn Community, INRIA and contributors), imbalanced-learn v0.12.x (developed by the imbalanced-learn Developers, part of the scikit-learn-contrib community)), pandas v2.2.x (pandas Development Team, NumFOCUS Foundation), and numpy v1.26.x (NumPy Developers, NumFOCUS Foundation).

See supplementary material for detailed ML algorithms and AI related data applied for this study.

## 3 Results

A total of 72 patients met the inclusion criteria, with a mean age of 60.5 ± 14.7 years (40% were male). Hypertension was present in 54.2% of patients, diabetes mellitus in 19.4%, and renal insufficiency in 15.3%. Laboratory results showed that 66.6% of patients (48/72) were positive for LAC, 40.2% for aCL IgG (29/72), 23.6% for aCL IgM (17/72), 36.1% for anti-β_2_-GPI IgG (26/72), and 26.3% for anti-β_2_-GPI IgM (19/72). Triple positivity was observed in 20.82% of the total population (15/72). Regarding thrombophilia, in patients with no thrombotic recurrence, 6 of them were positive for the FV Leiden mutation and 3 for the PT20210A mutation, all of them in heterozygosis. In the group with recurrence, 6 patients had the FV Leiden mutation in heterozygosis. No other thrombophilia was detected.

A comparison of clinical and demographic variables in patients with and without thrombosis recurrence is summarized in Table 1.

**Table 1:** Comparison of clinical and demographic variables in patients with and without thrombosis recurrence.

Variables	With recurrence	Without recurrence	*P* value
	*n* = 22	*n* = 50	
Age (y), mean ± SD (y)	63.5 ± 14.0	59.2 ± 14.9	.248
Male, *n* (%)	11 (50)	18 (36)	.265
Renal insufficiency, *n* (%)	6 (27)	5 (10)	.061
Obesity, *n* (%)	22 (44)	9 (41)	.807
Thrombophilia, *n* (%)	6 (27)	9 (18)	.563
Venous location, *n* (%)	17 (77)	30 (60)	.156
Arterial location, *n* (%)	6 (27)	20 (40)	.300
LAC, *n* (%)	18 (82)	30 (60)	.070
Smokers, *n* (%)	3 (14)	12 (24)	.319
Diabetes Mellitus, *n* (%)	4 (18)	10 (20)	.857
Hypertension, *n* (%)	11 (50)	28 (56)	.638
Dyslipidemia, *n* (%)	13 (59)	30 (60)	.942
aCL IgM, *n* (%)	5 (23)	12 (24)	.907
aCL IgG, *n* (%)	7 (32)	22 (44)	.332
anti-β2GPI IgM, *n* (%)	7 (32)	12 (24)	.488
anti-β2GPI IgG, *n* (%)	6 (27)	20 (40)	.300
Triple positivity, *n* (%)	5 (23)	10 (20)	.793
LMWH, *n* (%)	1 (4)	4 (8)	.595
DOAC, *n* (%)	2 (10)	1 (2)	.165
VKA, *n* (%)	19 (86)	45 (90)	.140
INR >60%	11 (58)	28 (62)	.649

aCL, anticardiolipin; DOAC, direct oral anticoagulants; Ig, immunoglobulin; LMWH, low-molecular-weight heparin; INR, international normalized ratio; LAC, lupus anticoagulant; VKA, vitamin K antagonists.

A comprehensive comparison of model performance metrics is presented in Table 2. Among the ML algorithms evaluated, XGB demonstrated superior performance during the testing phase. Across 200 repeated nested CV runs, performance estimates for AUC-ROC, PR (average precision)-AUC, balanced accuracy, and Brier score were stable with narrow confidence intervals (Supplementary Table 2). Importantly, no outer CV iteration exhibited a collapse in discrimination or calibration. When compared with alternative classifiers—including regularized logistic regression, decision tree, K-nearest neighbors, Gaussian naïve Bayes, and Random Forest—XGB consistently demonstrated modest yet reproducible performance advantages, without evidence of instability. Model performance was consistent across all imbalance-handling strategies. Differences in AUC-ROC between SMOTE and alternative methods (Borderline-SMOTE, SMOTEENN, and cost-sensitive learning) did not exceed 0.02, and PR (average precision)-AUC values overlapped across approaches. Decision-curve analyses likewise showed nearly identical net benefit profiles, indicating that the discriminative ability and clinical utility of the models were robust to the specific class-balancing technique employed. An ablation analysis compared pipelines with and without winsorization and scaling, and with and without collinearity refinement. Discrimination and calibration were consistent across configurations. Scaling modestly benefited margin- and distance-based classifiers such as Support Vector Machine and k-NN, whereas XGB and Random Forest were largely unaffected. Collinearity refinement reduced variance and improved calibration in linear models but did not alter conclusions for tree-based approaches. Decision-curve analyses demonstrated overlapping net benefit across preprocessing variants.

**Table 2:** Performance metrics achieved during the training and testing phases of the machine learning models.

Training phase								
ML alogrithm	Accuracy (%)	Recall (%)	Specificity (%)	Precision (%)	DYI (%)	F1 score (%)	MCC (%)	AUC-ROC (%)
SVM	83.5	83.6	83.4	82.9	83.5	83.3	74.1	83.3
DT	81.7	81.8	81.6	81.2	81.7	81.5	72.5	81.9
GNB	78.7	78.8	78.6	78.1	78.7	78.5	69.8	78.2
KNN	86.2	86.3	86.1	85.6	86.2	86.0	76.5	86.4
RF	87.6	87.5	87.7	87.0	87.6	87.3	78.9	87.8
XGB	91.9	92.0	91.8	91.3	91.9	91.6	81.6	92.0

Testing phase								
ML alogrithm	Accuracy (%)	Recall (%)	Specificity (%)	Precision (%)	DYI (%)	F1 score (%)	MCC (%)	AUC-ROC (%)
SVM	82.4	82.5	82.3	81.8	82.4	82.2	73.1	82.3
DT	80.6	80.7	80.6	80.1	80.6	80.4	71.6	80.5
GNB	77.5	77.6	77.4	76.9	77.5	7.2	68.7	77.3
KNN	85.1	85.2	85.0	84.5	85.1	84.8	75.5	85.0
RF	87.2	87.3	87.1	86.8	87.2	87.0	79.5	87.0
XGB	90.9	91.0	90.8	90.3	90.9	90.6	80.7	91.0

AUC-ROC, area under the receiver operating characteristic curve; DT, decision tree; DYI, Youden’s Index; GNB, Gaussian Naïve Bayes; KNN, K-nearest neighbors; MCC, Matthews correlation coefficient; RF, random forest; SVM, support vector machine; XGB, extreme gradient boosting.

**Figure 1:**
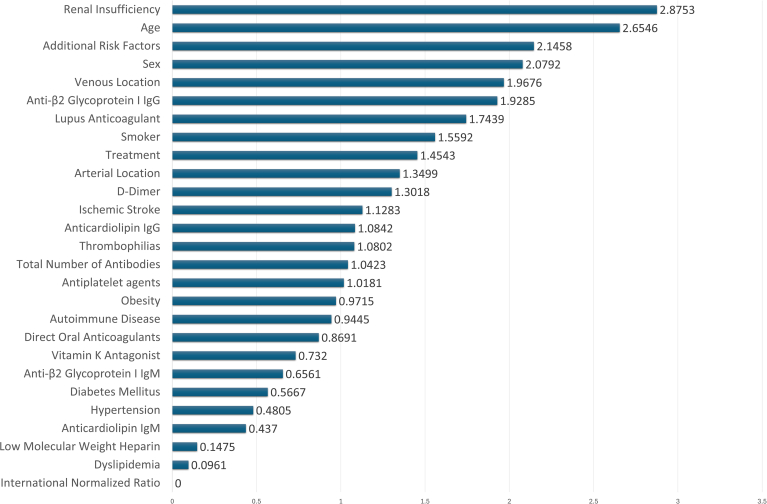
Variable and weights assigned to each variable, indicating their relative contribution to the model.

**Figure 2:**
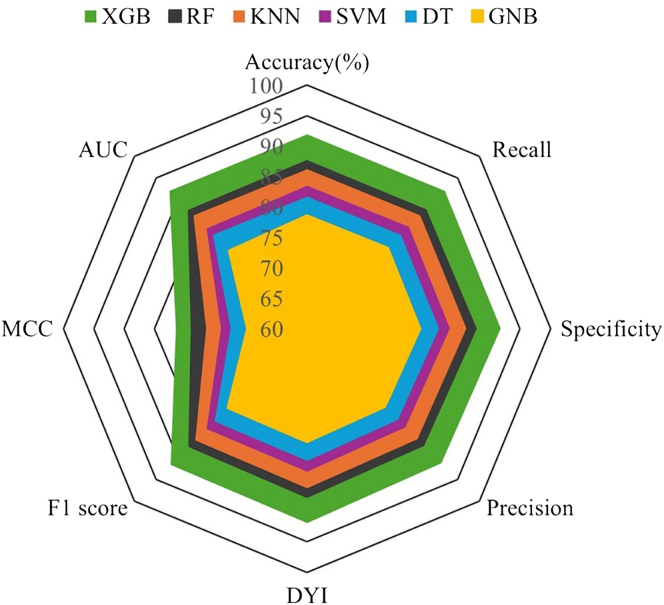
Radar Chart of machine learning algorithm performance during the training phase. AUC, area under the curve; DT, decision tree; DYI, Youden’s Index; GNB, Gaussian naïve Bayes; KNN, K-nearest neighbors; MCC, Matthews correlation coefficient; RF, random forest; SVM, support vector machine; XGB, extreme gradient boosting.

**Figure 3:**
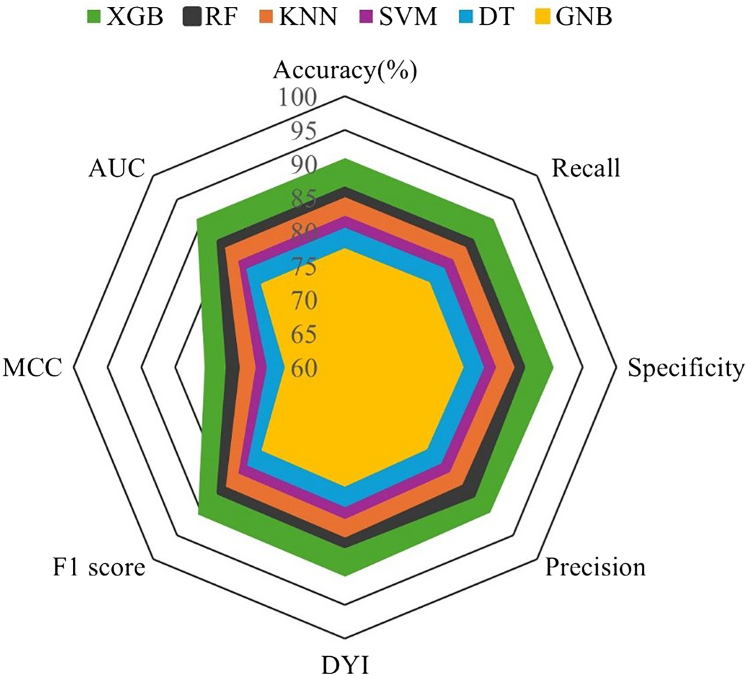
Radar Chart of machine learning algorithm performance during the testing phase. AUC, area under the curve; DT, decision tree; DYI, Youden’s Index; GNB, Gaussian naïve Bayes; KNN, K-nearest neighbors; MCC, Matthews correlation coefficient; RF, random forest; SVM, support vector machine; XGB, extreme gradient boosting.

**Figure 4:**
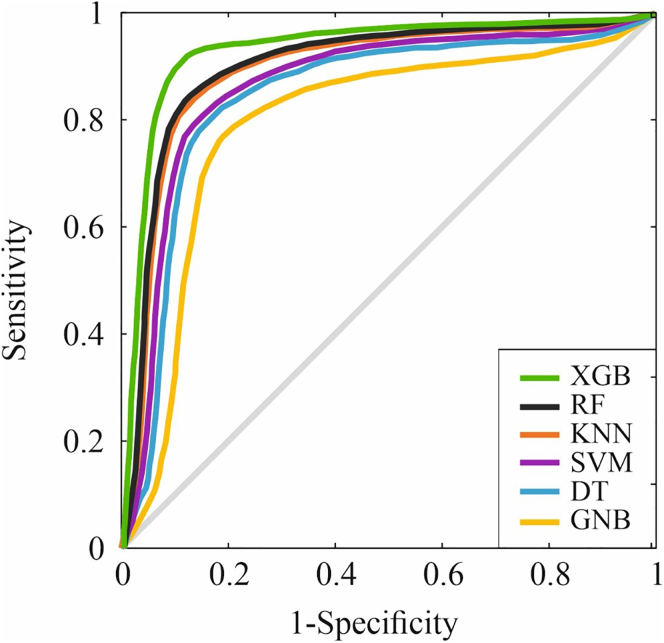
A multi-model ROC curve comparing the AUC-ROC of all 5 algorithms. DT, decision tree; GNB, Gaussian naïve Bayes; KNN, K-nearest neighbors; RF, random forest; SVM, support vector machine; XGB, extreme gradient boosting.

The relative contributions of the study variables are illustrated in Figure 1 (Supplementary Table 3 for additional details). The feature importance analysis of XGB identified renal insufficiency, LAC, venous location, and age as the most significant predictors of thrombotic recurrence. XGB achieved an AUC-ROC of 0.9125, an F1-score of 91.24, and a Mathews correlation coefficient of 80.98, with recall and accuracy exceeding 92.23% and 91.35%, respectively, highlighting its robust predictive capability compared with other models. See Figure 2, Figure 3, Figure 4.

## 4 Discussion

The findings of this study enhance the potential of ML, particularly XGB, in predicting recurrent thrombotic events in patients with TAPS. XGB outperformed traditional predictive models by effectively integrating clinical, demographic, and laboratory variables to generate accurate risk assessments. Its ability to handle heterogeneous data, detect complex interactions among multiple variables, and mitigate overfitting through regularization contributes to its superior predictive capabilities [[25], [26], [27]].

The identification of renal insufficiency, age, and LAC as key predictors aligns with existing clinical knowledge, reinforcing the understanding that renal dysfunction exacerbates endothelial damage and significantly contributes to thrombotic risk [28]. Age, a well-established risk factor, is associated with physiological changes such as arterial stiffening, endothelial dysfunction, and increased coagulability, all of which increase thrombotic risk [29]. Additionally, aging is linked to enhanced activation of the coagulation cascade and elevated levels of pro-inflammatory biomarkers that promote thrombosis [30]. Older patients frequently present with comorbidities, including hypertension, diabetes, and dyslipidemia, which further contribute to vascular dysfunction and increase the likelihood of recurrent thrombotic events [31]. The presence of LAC has been identified as the most potent thrombotic risk factor in APS, significantly increasing the risk of arterial and venous thrombosis [12]. The presence of LAC in patients with TAPS has been associated with a significant increase in resistance to activated protein C, a key mechanism in coagulation regulation [32]. This resistance decreases activated protein C ability to inactivate factors Va and VIIIa, promoting a prothrombotic state due to higher levels of thrombin generation after coagulation activation. Excess thrombin subsequently activates thrombin-activatable fibrinolysis inhibitor, an enzyme that stabilizes the clot by removing plasminogen-binding sites on fibrin, thereby reducing plasminogen conversion into plasmin and blocking fibrinolysis [33]. In addition, enhanced thrombin generation also induces the production of plasminogen activator inhibitor-1 [34], a potent fibrinolysis suppressor that prevents the activation of plasminogen by tissue plasminogen activator [35]. Elevated plasminogen activator inhibitor-1 levels further contribute to fibrin accumulation and persistent clot formation. Together, these mechanisms generate a prothrombotic state in TAPS patients, explaining the role of LAC as a key risk factor for arterial and venous thrombosis in this population. In addition to the presence of LAC, the presence of aCL and anti-β_2_-GPI antibodies are contributing variables to our predictive model, which aligns with previous findings indicating that triple positivity has been strongly associated with a higher likelihood of recurrent thrombotic events in TAPS patients [9].

A significant observation in this study is that the INR did not exhibit predictive value for thrombotic recurrence. This may be attributed to the presence of confounding factors not captured by the model or that INR alone is not a reliable predictor of thrombotic events [[36], [37], [38]]. The INR distribution in our cohort was relatively narrow, as most patients were under close clinical follow-up and maintained within therapeutic ranges, leaving limited variability to inform recurrence risk. In addition, transient INR fluctuations may be more relevant to immediate bleeding or thrombosis risk than to long-term recurrence, which appears to be better captured by stable predictors such as comorbidities, renal function, or inflammatory markers. While INR is a standard tool for monitoring anticoagulation with VKA, recent studies suggest that point of care INR testing may not reflect the real anticoagulation activity of VKA in APS, as interactions between antiphospholipid antibodies and thromboplastin reagent may occur. This finding may explain its limited utility in our predictive model [39,40]. Masucci et al. [41] described 87.2% of paired point of care and venous INR in 36 APS patients, with high correlation (r > 0.9), but excluding INR ≥ 4.8. The main limitation of this study relies on the sample size, although recent evidence suggests that ML models can achieve reliable performance even in small cohorts when appropriate validation techniques, such as CV, are applied [42]. To further mitigate the impact of the limited sample size, we specifically applied the SMOTE to balance recurrence and non-recurrence cases. SMOTE was applied strictly within the training folds of the nested CV pipeline, after imputation and scaling but before model training, thereby avoiding any leakage of synthetic cases into the test folds. Sensitivity analyses using alternative approaches, including Borderline-SMOTE, SMOTEENN, and cost-sensitive learning, confirmed that discrimination and clinical utility were stable across methods, supporting the robustness of our conclusions. To improve predictive performance, future research should consider incorporating additional aPL, such as anti-phosphatidylserine/prothrombin and anti-annexin-V, which have demonstrated strong associations with thrombotic events in recent studies [43,44]. Furthermore, platelet lipidomic may offer valuable insights into thrombotic risk stratification [45,46]. In addition, further external validation requires larger, multicenter cohort design and diverse populations, which could also improve the accuracy and applicability of the predictive models and contribute to generalizability. From a clinical perspective, the adoption of ML tools like XGBoost has the potential to revolutionize patient care by enabling personalized risk stratification and optimizing anticoagulation strategies. However, for successful real-world implementation, user-friendly interfaces and integration into electronic health record systems will be essential to facilitate clinical decision-making.

## 5 Conclusion

This study demonstrates the potential of XGB in TAPS management, highlighting its robust predictive capability compared with other models. Key predictors such as renal insufficiency, age, and LAC highlight the importance of integrating clinical and laboratory data for an accurate risk stratification. Integrating multi-omics data, such as platelet lipidomic, and additional aPL, like anti-phosphatidylserine/prothrombin and anti-annexin-V, could boost predictive accuracy by uncovering molecular interactions and pathways driving thrombotic risk in platelets, the main target of aPL antibodies. These approaches provide a comprehensive view of the biological processes underlying TAPS, enabling the identification of novel biomarkers and therapeutic targets, ultimately improving patient outcomes and resource allocation in TAPS care.
