# Supplementary material for: Machine learning for predicting thrombotic recurrence in antiphospholipid syndrome
Source: Res Pract Thromb Haemost. 2025 Sep 30;9(7):103198. doi: 10.1016/j.rpth.2025.103198 (PMC12616068; doi:10.1016/j.rpth.2025.103198)
Supplement: Supplementary Material [file mmc1.docx]

**Supplementary material.**

Machine Learning (ML) algorithms are mainly divided into four categories: supervised learning, unsupervised learning, semi-supervised learning, and reinforcement learning.

-Supervised learning aims to learn a function that maps an input to an output based on sample input-output pairs. Supervised learning is carried out when certain goals are identified to be accomplished from a certain set of inputs.

-Unsupervised learning analyzes unlabeled datasets without the need for human intervention. It is used for extracting generative features, identifying meaningful trends and structures, groupings in results, and exploratory purposes.

-Semi-supervised learning operates on both labeled and unlabeled data. The ultimate goal of this model is to provide a better outcome for prediction than that produced using the labeled data alone from the model. Some application areas where semi-supervised learning is used include machine translation, fraud detection, labeling data and text classification.

-Reinforcement learning enables software agents and machines to automatically evaluate the optimal behavior in a particular context or environment to improve its efficiency. This type of learning is based on reward or penalty, and its ultimate goal is to use insights obtained from environmental activists to take action to increase the reward or minimize the risk.

A general structure of a ML-based predictive model is based on 2 phases: Phase 1, the model is trained from historical data (training phase); and Phase 2, the outcome is generated from the new test data (testing phase).

A brief description of the ML classification algorithms used in this study is provided here.

***Extreme Gradient Boosting***, known as XGBoost (XGB), is a supervised, scalable and optimized algorithm that improves the prediction performance of Gradient Boosting Machines. For this purpose, this algorithm uses a new tree learning algorithm and leverages parallel and distributing computing to accelerate model discovery. XGBoost is fast to interpret and can handle large-sized datasets well. Gradient Boosting is an ensemble learning method used for classification and regression tasks. This model is trained to minimize loss function such as mean squared error of the previous model using gradient descent. In each iteration, the algorithm computes the gradient of the loss function with respect to predictions and then trains a new model to minimize this gradient. Predictions are then added to the ensemble (all models prediction) and the process is repeated until a stopping criterion is met.

***Random Forest*** (RF) is a commonly used supervised [ML](https://www.ibm.com/topics/supervised-learning) algorithm that manages many decision trees to make better predictions. Each tree looks at different random parts of the data and are combined by voting for classification or averaging regression. Since RF algorithms can handle large data sets, they can provide more accurate predictions, but can be slow to process data as they are computing data for each individual decision tree. RF reduces the risk of overfitting.

***Support vector machine*** (SVM) is a supervised [ML](https://www.ibm.com/topics/supervised-learning) algorithm that classifies data by finding an optimal line that maximizes the distance between each class in an N-dimensional space to find the best decision boundary between classes. This enables to generalize well to new data and make accurate classification predictions. The lines that are adjacent to the optimal hyperplane are known as support vectors as these vectors run through the data points that determine the maximal margin. The SVM algorithm is widely used in [ML](https://www.ibm.com/think/topics/machine-learning) as it can handle both linear and nonlinear classification tasks. However, when the data set contains more noise, such as overlapping target classes, SVM does not perform well.

***Decision tree*** is a non-parametric supervised learning algorithm, which is utilized for both classification and regression tasks. It has a hierarchical, tree structure, which consists of a root node, branches, internal nodes and leaf nodes. A decision tree starts with a root node. The outgoing branches from the root node then feed into the internal nodes, also known as decision nodes. Both node types conduct evaluations to form homogenous subsets, which are denoted by leaf nodes, or terminal nodes. The leaf nodes represent all the possible outcomes within the dataset.

***Gaussian Naive Bayes*** is a supervised classification ML algorithm based on the probabilistic approach and Gaussian distribution (normal distribution). It assumes that each parameter has an independent capacity of predicting the output variable. The combination of the prediction for all parameters is the final prediction that returns a probability of the dependent variable to be classified in each group. The final classification is assigned to the group with the higher probability. The main advantage, compared to more sophisticated approaches, is the need of a small amount of training data to estimate the necessary parameters. However, its performance may affect due to its strong assumptions on features independence.

***K-Nearest Neighbors (KNN)*** is a non-parametric supervised ML algorithm generally used for classification but can also be used for regression tasks. It works by finding the "k" closest data points (neighbors) to a given input and makes a prediction based on the majority class (for classification) or the average value (for regression). The biggest issue with KNN is to choose the optimal number of neighbors to be considered.

Other specific data that appear in this manuscript are:

1. *Recall,* also called sensitivity, measures the model's ability to detect positive events correctly. It is the percentage of accurately predicted positive events out of all actual positive events.
2. Area Under the Receiver Operating Characteristic Curve (AUC-ROC) represents the probability that the model, if given a randomly chosen positive and negative example, will rank the positive higher than negative.
3. Youden's Index is a statistical measure used to assess the effectiveness of a diagnostic test. It is calculated as the maximum vertical distance between the curve of true positive rates (sensitivity) and false positive rates (1-specificity) on a ROC curve This index helps determine the optimal cut-off point for a test, balancing sensitivity and specificity, making it essential for evaluating diagnostic performance.
4. The *F1 Score* is described as the harmonic mean of the precision and recall of a classification model. The two metrics contribute equally to the score, ensuring that the F1 metric correctly indicates the reliability of a model.
5. The *Matthews Correlation Coefficient* (MCC) is a measure of the quality of binary classifications. It takes into account all four elements of a confusion matrix: true positives, true negatives, false positives, and false negatives. The MCC can be understood as a correlation coefficient between the predicted and actual classifications, ranging from -1 to +1. An MCC of +1 indicates perfect predictions, 0 indicates no better than random guessing, and -1 indicates total disagreement between predictions and true outcomes

**Supplementary Table 1.**

*Comparative performance of imbalance-handling strategies.*

Summary of discrimination and performance metrics for XGB models trained with different imbalance-handling methods. Values represent averages across 200 repeated nested cross-validation runs, with 95% confidence intervals where applicable. Results were consistent across methods, confirming that the observed predictive performance and clinical utility are robust to the specific imbalance-mitigation technique applied.

| Method | ROC-AUC (95% CI) | PR-AUC (95% CI) | Balanced Accuracy (%) | MCC (%) | Brier Score |
| --- | --- | --- | --- | --- | --- |
| SMOTE | 0.910 (0.890–0.929) | 0.718 (0.697–0.738) | 90.9 | 80.7 | 0.162 |
| Borderline-SMOTE | 0.906 (0.886–0.925) | 0.712 (0.691–0.732) | 90.2 | 79.5 | 0.165 |
| SMOTEENN | 0.903 (0.883–0.922) | 0.710 (0.689–0.730) | 89.8 | 78.9 | 0.167 |
| Cost-sensitive XGB | 0.908 (0.888–0.927) | 0.716 (0.696–0.736) | 90.5 | 80.1 | 0.163 |

**Imbalance handling methods**: 1) SMOTE generates synthetic minority instances until class balance (1:1 ratio) is achieved, using five nearest neighbors for interpolation and applied only after imputation and scaling had been fitted on the training data; 2) Borderline-SMOTE, which preferentially synthesizes borderline minority samples; 3) SMOTEENN, which combines oversampling with cleaning of ambiguous or mislabeled cases; 4) Cost-sensitive learning in XGB, implemented via adjustment of the scale_pos_weight parameter to match the observed negative-to-positive ratio.

Note: PR-AUC values are reported as Average Precision (AP). The baseline AP equals the prevalence of recurrence in the cohort (0.306). Thus, PR-AUC values ≈0.70 represent strong discriminative performance despite class imbalance.

**Supplementary Table 2.**

*Ablation analysis of preprocessing and collinearity refinement.*

Comparison of model performance under four alternative preprocessing pipelines: (i) full preprocessing with winsorization, scaling, and collinearity refinement; (ii) without winsorization/scaling; (iii) without collinearity refinement; and (iv) without both. Results are averaged across 200 repeated nested cross-validation runs. Discrimination (ROC-AUC, PR-AUC) and calibration (Brier score, calibration slope) were consistent across variants, with differences remaining within narrow confidence intervals. Tree-based models (XGB, RF) were largely insensitive to preprocessing choices, whereas linear and distance-based classifiers showed modest improvements with scaling and collinearity refinement.

| Pipeline Variant | ROC-AUC (95% CI) | PR-AUC (95% CI) | Brier Score | Calibration slope |
| --- | --- | --- | --- | --- |
| Full preprocessing (winsorization + scaling + collinearity refinement) | 0.910 (0.890–0.929) | 0.718 (0.697–0.738) | 0.162 | 0.99 |
| Without winsorization/scaling | 0.907 (0.887–0.926) | 0.714 (0.693–0.734) | 0.165 | 0.95 |
| Without collinearity refinement | 0.905 (0.885–0.924) | 0.712 (0.691–0.732) | 0.166 | 0.96 |
| Without winsorization/scaling + without collinearity refinement | 0.902 (0.882–0.921) | 0.710 (0.689–0.730) | 0.168 | 0.94 |

While ROC-AUC values were close to 0.91, PR-AUC values were lower (~0.71). This difference is expected in imbalanced datasets, as PR-AUC is directly influenced by prevalence (baseline = 0.306 in our cohort). Thus, a PR-AUC of ~0.71 represents strong discriminative ability, more than doubling the baseline precision–recall performance.

# **Supplementary table 3.**

# *SHAP-based feature importance.*

The table reports the mean absolute SHAP value for each predictor, averaged across 200 repeated nested cross-validation runs. Variables are ranked in descending order of contribution to recurrence risk prediction. SHAP values quantify the average impact of each variable on the model’s output, with higher values indicating greater influence.

| Rank | Predictor | Mean absolute SHAP value | Direction of effect (summary) |
| --- | --- | --- | --- |
| 1 | Age | 0.085 | Higher age → increased recurrence risk |
| 2 | Creatinine | 0.072 | Higher values → increased risk |
| 3 | Diabetes mellitus | 0.060 | Presence → increased risk |
| 4 | Hypertension | 0.054 | Presence → increased risk |
| 5 | C-reactive protein (CRP) | 0.051 | Higher values → increased risk |
| 6 | Platelet count | 0.048 | Lower values → increased risk |
| 7 | Hemoglobin | 0.043 | Lower values → increased risk |
| 8 | Sex (male) | 0.039 | Male sex → increased risk |
| 9 | Smoking status | 0.035 | Current smoking → increased risk |
| 10 | INR | 0.012 | Minimal contribution |

Note: SHAP = SHapley Additive exPlanations. Variables are ranked by mean absolute SHAP values across all predictions. Direction of effect is derived from SHAP summary plots (Supplementary Figure 1). INR, although clinically relevant, showed minimal impact in this dataset.

**References.**

1. Sarker IH. Machine learning: algorithms, real-world applications and research directions. SN Comput Sci 2021; 2: 160.
2. Mahesh B, Machine learning algorithms: a review. Int J Sci Res 2018; 9:381-386.
3. Rainio O, Teuho J, Klen R. Evaluation metrics and statistical tests for machine learning. Sci Rep 2024; 14:6086.
